# Supplementary material for: Quantum localization and delocalization of charge carriers in organic semiconducting crystals
Source: Nat Commun. 2019 Aug 26;10:3843. doi: 10.1038/s41467-019-11775-9 (PMC6710274; doi:10.1038/s41467-019-11775-9)
Supplement: Supplementary file 1 — Supplementary Information [file 41467_2019_11775_MOESM1_ESM.pdf]

Supplementary Information: Quantum Localization and Delocalization of Charge Carriers in Organic Semiconducting Crystals

S. Giannini *et al.*

Supplementary Tables

Supplementary Table 1: Crystal structures and couplings comparison.

crystal	dir.	dist. (Å)	H_{kl} (lit.) (meV)	H_{kl}(FODFT) ^{r} (meV)
DATT-h^{+} ^{a}	a	6.26	70^{a}, 66.8^{o}, 86.5^{p}	94.9
RUB-h^{+} ^{b}	a	7.18	81^{i}, 83^{j}, 107^{k}, 140^{l}, 95.7^{m}	113.4
PEN-h^{+} ^{c}	T_1	4.80	61^{i}, 85^{j}, 81^{k}, 130.6^{l}, 96.7^{n}, 90.7^{m}	116.1
ANT-h^{+} ^{d}	a	5.24^{q}	26^{i}, 23^{j}	17.6
	b	6.04	40^{i}, 44^{j}	57.2
NAP-h^{+} ^{e}	b	5.95	43^{i}, 35^{j},	46.2
PER-e^{-} ^{f}	a	6.10^{q}	-	61.7
	c^{*}	10.26	-	8.3
PYR-e^{-} ^{g}	c^{*}	8.47	-	26.7
pMSB-h^{+} ^{h}	b	5.88	-	21.5

^{a} Ref.[1] ^{b} Ref.[2] ^{c} Ref.[3] ^{d} Ref.[4] ^{e} Ref.[5] ^{f} Ref.[6] ^{g} Ref.[7] ^{h} Ref.[8] ^{i} Ref.[9] ^{j} Ref.[10] ^{k} Ref.[11] ^{l} Ref.[12] ^{m} Ref.[13] ^{n} Ref.[14] ^{o} Ref.[15] ^{p} Ref.[16] ^{q} T-shaped molecular pair along the given direction. ^{r} Electronic couplings for crystal structure geometries obtained using scaled FODFT as described in *Molecular model* section.

Supplementary Figures

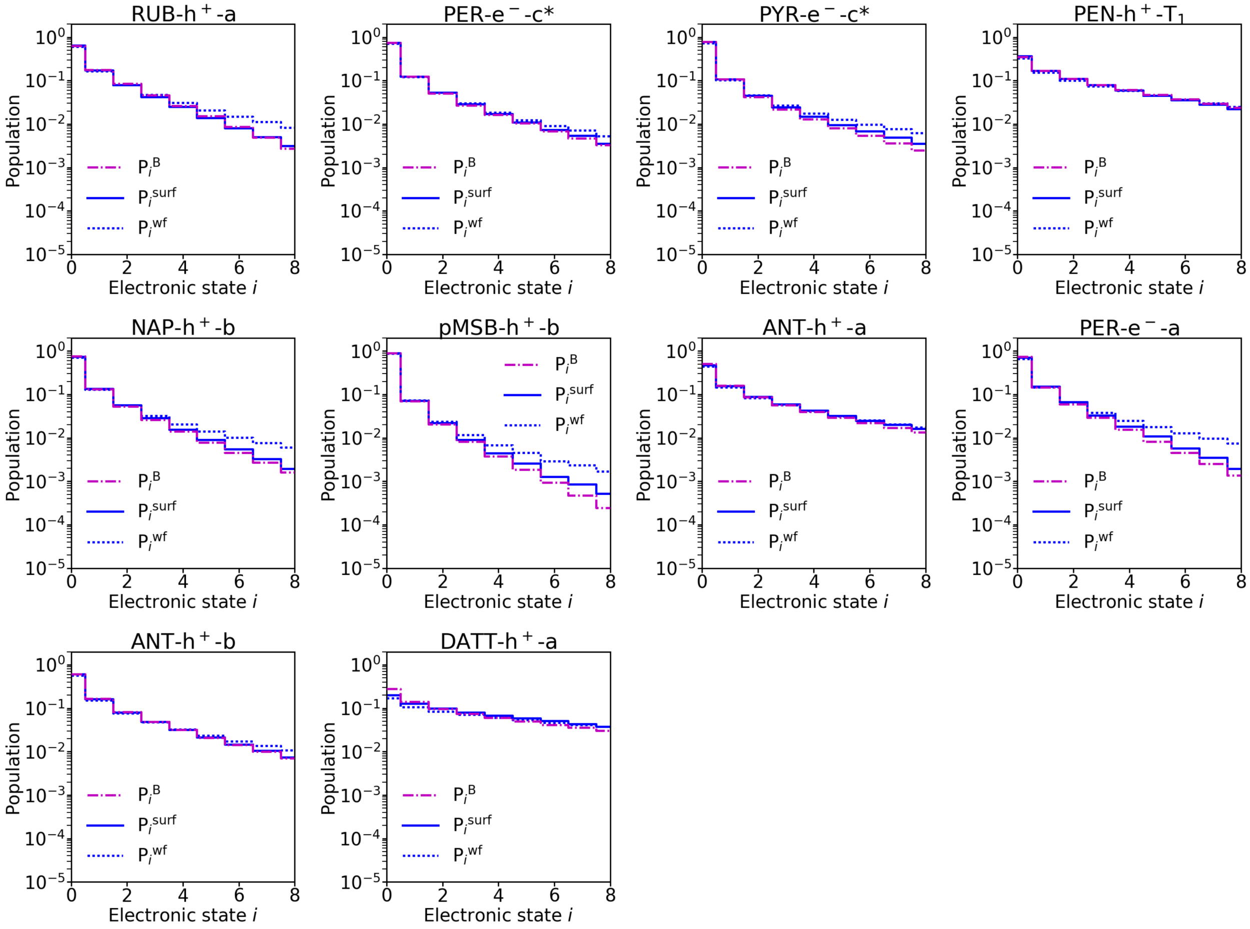

Supplementary Figure 1: Detailed balance and internal consistency in FOB-SH. the OS materials studied are reported in Fig. 1 main text. Electronic eigenstate populations of the carrier wavefunction $\Psi(t)$ (blue dotted) and of the active surface that determines the nuclear dynamics in surface hopping (solid blue) are obtained from an average over 1000 FOB-SH trajectories. FOB-SH simulations are carried out in the standard set-up as described in the main text including decoherence correction, state tracking and projection algorithm. The reference Boltzmann population of the electronic eigenstates (magenta) are calculated as detailed in Methods from 1000 equilibrium MD trajectories of 1 ps length.

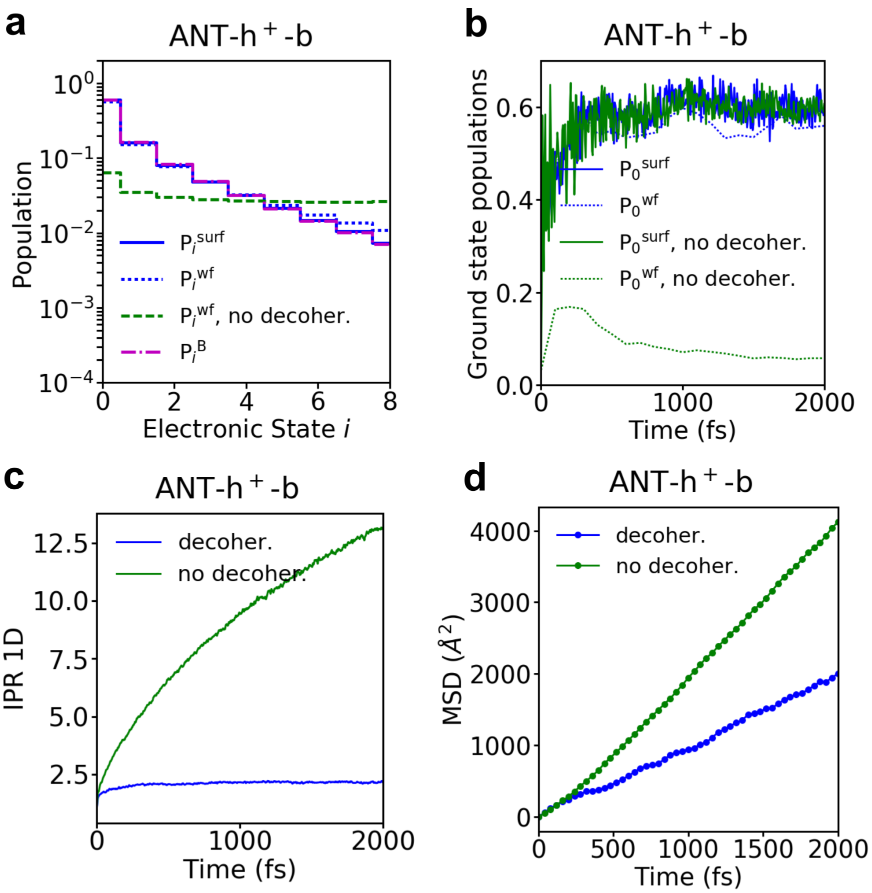

Supplementary Figure 2: Importance of decoherence correction for the charge transport. Illustrative FOB-SH hole transport trajectories have been carried out for anthracene crystal (ANT). (a) Electronic eigenstate (wf) and active surface (surf) population with decoherence correction (data in blue, from Supplementary Figure 1) and without decoherence correction (green dashed). In the latter case the electronic eigenstates (also denoted adiabatic states) are almost equally populated, i.e. the electronic temperature tends to infinity, the infamous problem of the original surface hopping and Ehrenfest methods. (b) Ground state population vs time with decoherence (blue) and without decoherence correction (green). Note that, without decocherence, despite the surface population reaches the correct equilibrium distribution after 300 fs, the ground state population remains severely underestimated. (c) Convergence of IPR with and without decoherence correction (blue and green lines). The IPR diverges without decoherence correction because of the near equal population of all electronic eigenstates (panel b). (d) MSD of the charge carrier (see Methods), with and without decoherence correction (blue and green lines). Without decoherence the slope of the MSD and mobility are strongly overestimated.

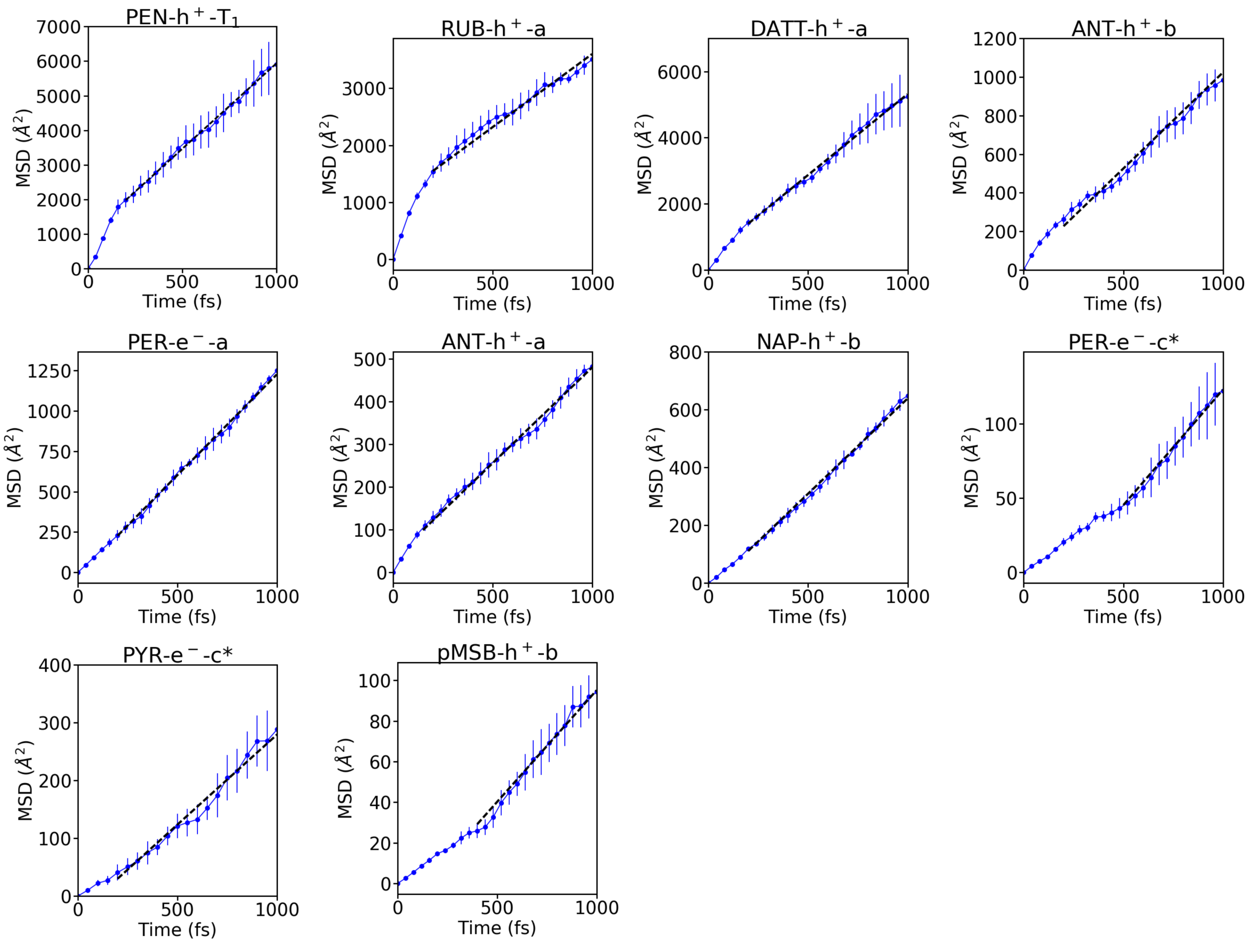

Supplementary Figure 3: Mean square displacement (MSD) of the charge carriers. MSD is obtained from 1000 FOB-SH trajectories of the OS materials shown in Fig. 1 (data in blue). Error bars are obtained by block-averaging over 5 blocks, 200 trajectories each. The diffusion coefficient is obtained from linear fits to the data from about 0.5 to 1 ps (dashed black lines).

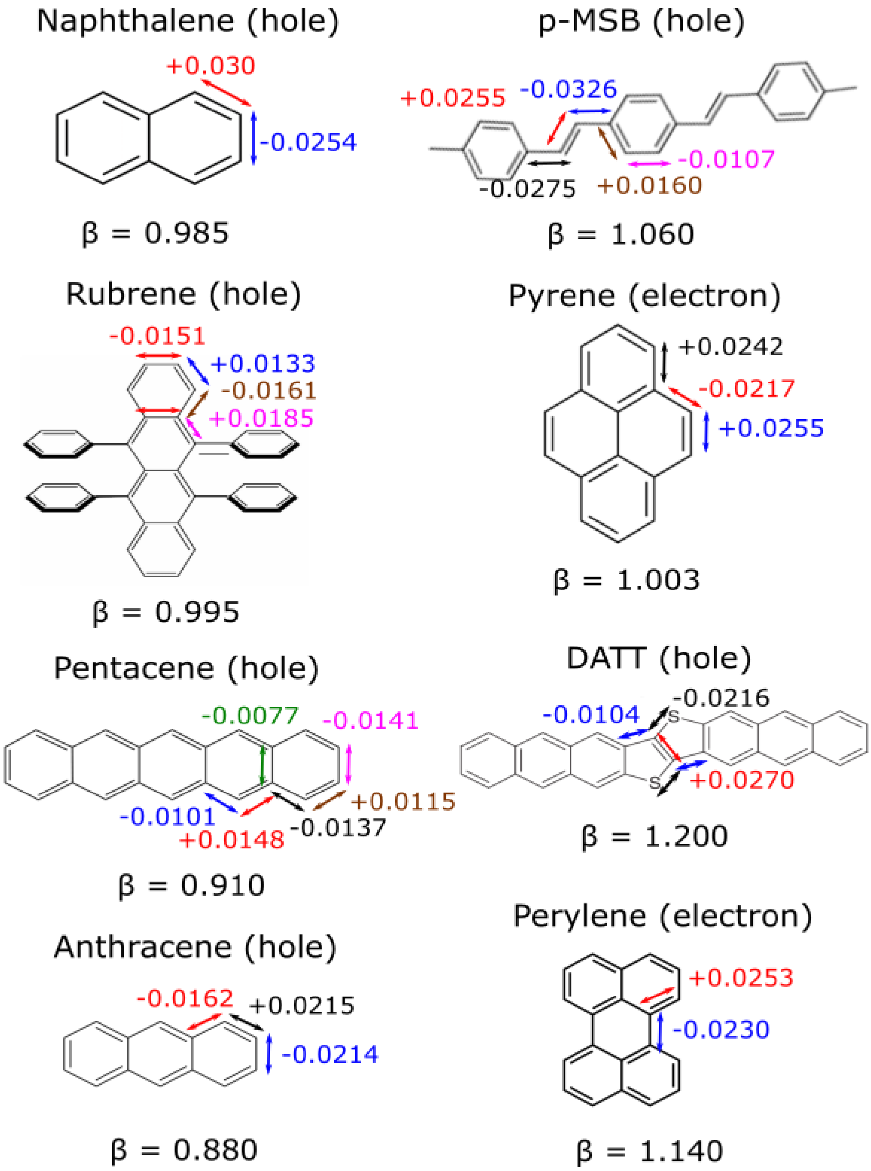

Supplementary Figure 4: Force-field parametrization. Changes in bond length upon change from neutral to charged state, as obtained from DFT calculations. Displaced bond distances in Å are reported with different colours according to the displaced bonds. The + and − signs indicate an increase and decrease in bond length going from the neutral to the charged system, respectively. The displacements are used to parametrize the force field for the molecules in their charged state. For clarity, displacements symmetric to the ones indicated are not shown. Scaling factor β for force field parametrization of reorganization energy, as described in Methods section, is also reported for each system.

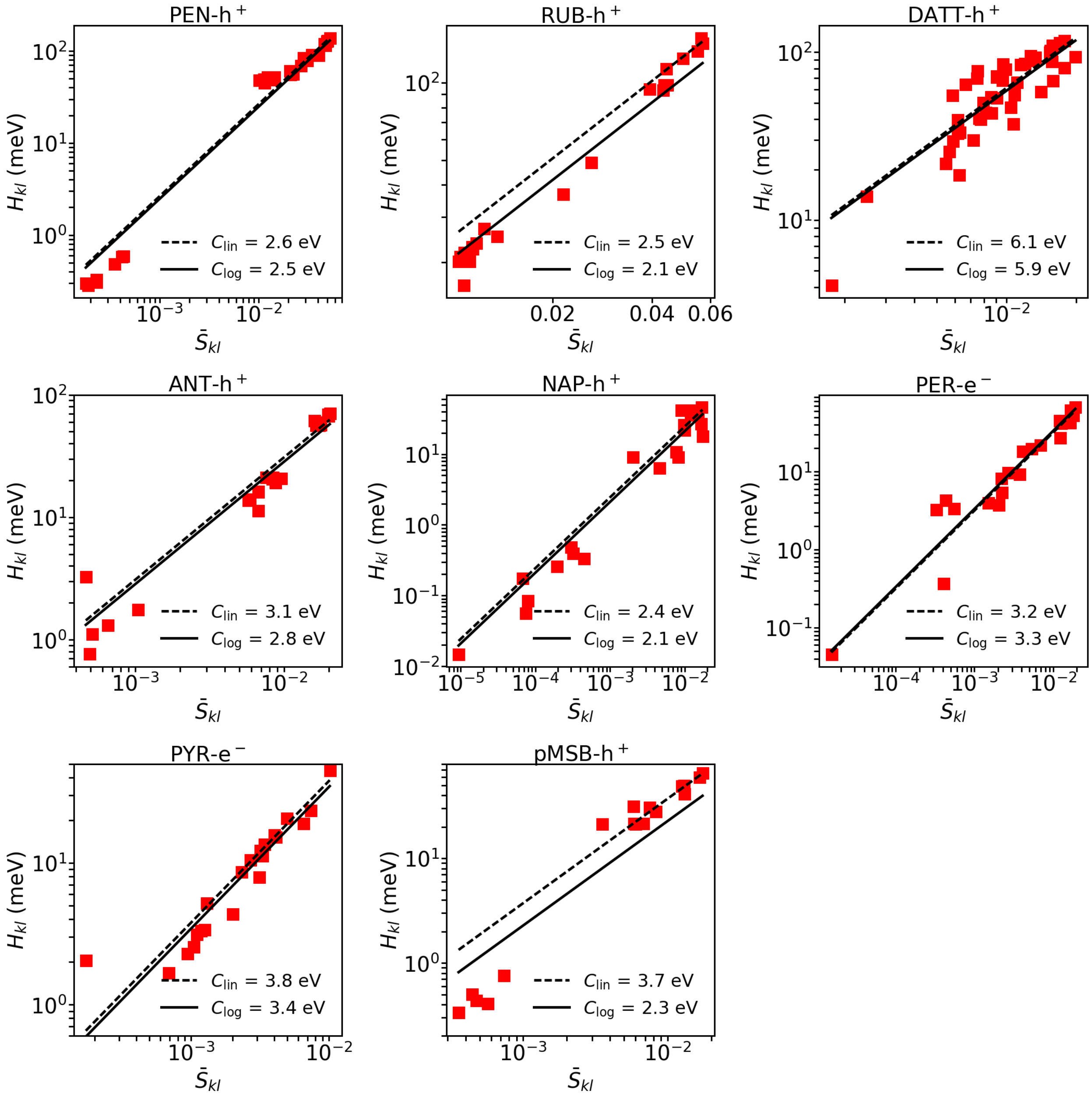

Supplementary Figure 5: Analytic overlap method (AOM) couplings parametrization. Electronic couplings H_{kl} between neighbouring molecules in the crystal structure and along MD trajectories were obtained from FODFT[17,18] calculations, scaled by a factor of 1.348 and 1.325 for hole and electron transfer, respectively, as recommended in Refs.[19,20] and plotted against the HOMO or LUMO overlap $\bar{S}_{kl}$. The constant of proportion C for the approximately linear relationship $H_{kl} = C\bar{S}_{kl}$ was obtained from linear (dashed) or logarithmic fitting (solid), giving $C = C_{\text{lin}}$ and $C = C_{\text{log}}$, respectively.

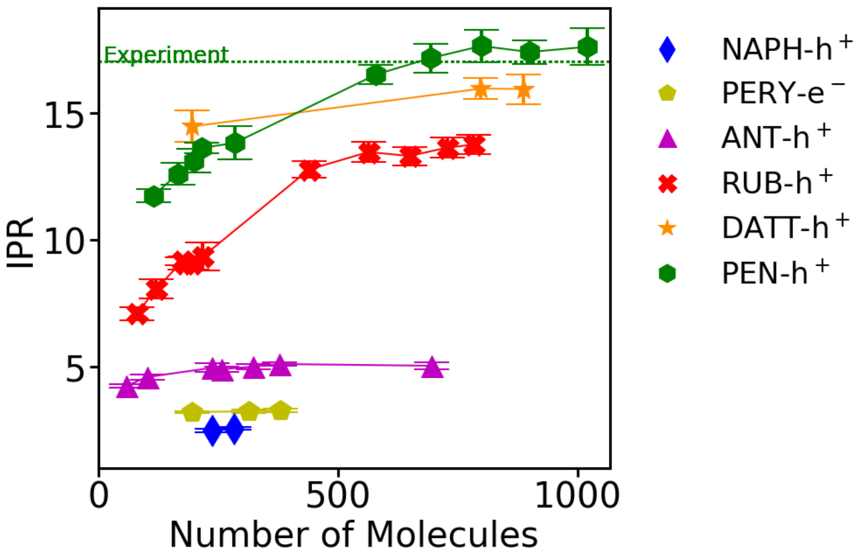

Supplementary Figure 6: Convergence of the IPR with respect to the number of molecules. Hole or electron transport IPR are reported for trajectories in the 2D herringbone layer of the systems indicated with different colors. The data were averaged over at least 200 FOB-SH trajectories and error bars were obtained by block averaging the equilibrated region of the IPR. Experimental wavefunction delocalization for Pentacene (Ref. 21) is given with green dotted line.

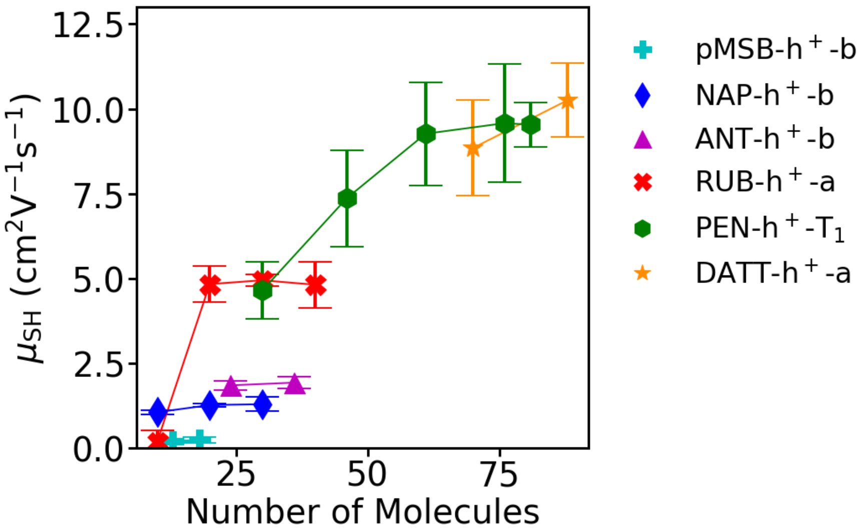

Supplementary Figure 7: Convergence of FOB-SH mobility with respect to the number of molecules. An active chain of molecules for different systems lengths and directions has been considered. The data were averaged over at least 600 FOB-SH trajectories and error bars were obtained by block averaging over blocks of 200 trajectories.

Supplementary References

1. Niimi, K., Shinamura, S., Osaka, I., Miyazaki, E. & Takimiya, K. Dianthra [2, 3-b: 2, 3-f] thieno [3, 2-b] thiophene (DATT): synthesis, characterization, and FET characteristics of new π-extended heteroarene with eight fused aromatic rings. *J. Am. Chem. Soc.* **133,** 8732–8739 (2011).

2. Bulgarovskaya, I., Vozzhennikov, V. & Aleksandrov, S. V. Bel skii, Latv. PSR Zinat. *Akad. Vestis Kim. Ser* **4,** 53 (1983).

3. Holmes, D., Kumaraswamy, S., Matzger, A. J. & Vollhardt, K. P. C. On the Nature of Nonplanarity in the [N] Phenylenes. *Chem.-Eur. J* **5,** 3399–3412 (1999).

4. Mason, R. The crystallography of anthracene at 95 K and 290 K. *Acta Crystallogr.* **17,** 547–555 (1964)

5. Ponomarev, V., Filipenko, O. & Atovmyan, L. CRYSTAL AND MOLECULAR-STRUCTURE OF NAPHTHALENE AT-150DEGREESC. *Kristallografiya* **21,** 392–394 (1976).

6. Camerman, A., Trotter, J. & Robertson, J. M. The crystal and molecular structure of perylene. *Proc. R. Soc. Lond.* **279,** 129–146 (1964)

7. Camerman, A. & Trotter, J. The crystal and molecular structure of pyrene. *Acta Crystallogr.* **18,** 636–643 (1965).

8. Kabe, R., Nakanotani, H., Sakanoue, T., Yahiro, M. & Adachi, C. Effect of molecular morphology on amplified spontaneous emission of bis-styrylbenzene derivatives. *Adv. Mat.* **21,** 4034–4038 (2009).

9. Yavuz, I., Martin, B. N., Park, J. & Houk, K. N. Theoretical study of the molecular ordering, paracrystallinity, and charge mobilities of oligomers in different crystalline phases. *J. Am. Chem. Soc.* **137,** 2856–2866 (2015).

10. Coropceanu, V. et al. Charge Transport in Organic Semiconductors. *Chem. Rev.* **107,** 926–952 (2007).

11. Kobayashi, H. et al. Hopping and band mobilities of pentacene, rubrene, and 2,7-dioctyl[1]benzothieno[3,2-b][1]benzothiophene (C8-BTBT) from first principle calculations. *J. Chem. Phys.* **139,** 014707 (2013).

12. Fratini, S., Ciuchi, S., Mayou, D., Laissardiere, G. T. d. & Troisi, A. A map of high-mobility molecular semiconductors. *Nat. Mater.* **16,** 998–1002 (2017).

13. Stehr, V., Pfister, J., Fink, R., Engels, B. & Deibel, C. First-principles calculations of anisotropic charge-carrier mobilities in organic semiconductor crystals. *Phys. Rev. B* **83,** 155208 (2011).

14. Sokolov, A. N. et al. From computational discovery to experimental characterization of a high hole mobility organic crystal. *Nat. Comm.* **2,** 437 (2011).

15. Jiang, Y. et al. Nuclear quantum tunnelling and carrier delocalization effects to bridge the gap between hopping and bandlike behaviors in organic semiconductors. *Nano. Horizons* **1,** 53–59 (2016).

16. Nan, G. & Li, Z. Influence of lattice dynamics on charge transport in the dianthra [2, 3-b: 2, 3-f]-thieno [3, 2-b] thiophene organic crystals from a theoretical study. *Physs Chem. Chem. Phys.* **14,** 9451–9459 (2012).

17. Oberhofer, H. & Blumberger, J. Revisiting electronic couplings and incoherent hopping models for electron transport in crystalline C_{60} at ambient temperatures. *Phys. Chem. Chem. Phys.* **14,** 13846–13852 (2012).

18. Gajdos, F. Oberhofer, H. Dupuis, M. & Blumberger, J. On the Inapplicability of Electron-Hopping Models for the Organic Semiconductor Phenyl-C61-butyric Acid Methyl Ester (PCBM). *J. Phys. Chem. Lett.* **4,** 1012–1017 (2013).

19. Kubas, A. et al. Electronic couplings for molecular charge transfer: benchmarking CDFT, FODFT and FODFTB against high-level ab initio calculations. *J. Chem. Phys.* **140,** 104105–21 (2014).

20. Kubas, A. et al. Electronic couplings for molecular charge transfer: benchmarking CDFT, FODFT and FODFTB against high-level ab initio calculations. II. *Phys. Chem. Chem. Phys.* **17,** 14342–14354 (2015).

21. Marumoto, K., Kuroda, S., Takenobu, T. & Iwasa, Y. Spatial Extent of Wave Functions of Gate-Induced Hole Carriers in Pentacene Field-Effect Devices as Investigated by Electron Spin Resonance. *Phys. Rev. Lett.* **97,** 256603 (2006).
